# Membranous nephropathy in Kimura disease: A case report and literature review on renal biopsy findings 

**DOI:** 10.5414/CNCS111774

**Published:** 2025-12-19

**Authors:** Motoki Murata, Kenichi Koga, Satoshi Yamaoka, Masako Hasebe, Chiho Fukushima, Chiaki Omiya, Kensei Yahata

**Affiliations:** Department of Nephrology, Osaka Red Cross Hospital, Osaka, Japan

**Keywords:** membranous nephropathy, Kimura disease, PLA2R

## Abstract

Kimura disease (KD) is a chronic benign granulomatous disease. Approximately 20% of patients with KD have renal disease. Membranous nephropathy (MN) is one of the main renal pathologies in KD; however, the underlying mechanism remains unknown. We herein present a 28-year-old male diagnosed with KD after biopsy of a left lower eyelid mass 11 years earlier. He visited our hospital with edema in the lower legs and scrotum. A blood test showed a serum creatinine level of 0.95 mg/dL and serum albumin level of 0.9 g/dL. Urinalysis revealed heavy proteinuria with mild hematuria. Renal biopsy showed spike formation by PAM staining and granular deposits of IgG and C3 in the glomerular basement membrane by direct immunofluorescence microscopy (IF). Electron microscopy revealed subepithelial electron-dense deposits (EDD). IF staining for the phospholipase A2 receptor (PLA2R) was positive in the glomerular basement membrane, leading to a diagnosis of PLA2R-associated MN. Our literature review on MN in KD included 14 cases, all of which exhibited subepithelial EDD, while subendothelial EDD was absent in 10. PLA2R staining was positive in 2 of the 3 cases examined. The results of this case and the literature review suggest the involvement of autoantibodies against podocyte antigens in the pathogenesis of MN in KD. Further studies are needed on these antigens.

## Introduction 

Kimura disease (KD) is a rare and chronic benign granulomatous disease characterized by the formation of indistinct masses in the subcutaneous soft tissues and lymph nodes of the head and neck region [1]. The prevalence of KD is higher among young Asian men than among individuals of other ethnicities. The histological features of KD include follicular hyperplasia, the infiltration of eosinophils, lymphocytes, and plasma cells, and vascular proliferation. Serology testing generally indicates elevated serum IgE levels, and peripheral eosinophilia is observed in a complete blood count [2]. Approximately 20% of patients with KD have renal diseases, with 60 – 80% presenting with nephrotic syndrome. Membranous nephropathy (MN) is one of the main renal pathologies associated with KD. Although the pathogenesis of KD is presumed to involve trauma, infection, IgE-mediated allergic reactions, and autoimmune mechanisms, that of nephropathy associated with KD remains unclear [3]. We herein report a case of MN associated with KD in which the phospholipase A2 receptor (PLA2R) antigen tested positive. 

## Case report 

A 28-year-old male was diagnosed with KD at the age of 17 years after biopsy of a left lower eyelid mass. He underwent tumor excision at 18 years, with no evidence of recurrence for nearly a decade. However, the mass recurred 17 months ago and was treated with a daily infusion of 250 mg methylprednisolone for 3 days, followed by oral PSL, which was tapered to a maintenance dose of 5 mg. The patient had discontinued treatment 10 months before presentation. 

Two months prior to his visit, a routine health check-up revealed 1+ proteinuria for the first time. He then developed edema in the lower legs and scrotum, prompting hospitalization. A physical examination revealed bilateral eyelid edema, a left lower eyelid mass, and significant bilateral lower leg edema. Blood biochemistry showed a serum albumin concentration of 0.9 g/dL, a serum creatinine concentration of 0.95 mg/dL, eGFR of 78.9 mL/min/1.73m^2^, and a blood urea nitrogen concentration of 13.5 mg/dL (Table 1). Hematological findings revealed a normal hemoglobin concentration and platelet count, a total white blood cell count of 9,280/µL, and an eosinophil percentage of 35% (normal range, 0 – 8.5%). The serum concentration of IgE was elevated at 1,335 IU/mL (normal range, 10 – 340 IU/mL), while other immunoglobulins were within normal limits. Serum complement (C3, C4, and CH50) levels and antinuclear antibody titers were normal. Serum PLA2R antibodies were negative. A urinalysis showed heavy proteinuria (7.22 g/gCr) with mild hematuria (10 – 19/high-power field). 

**Figure 1.**
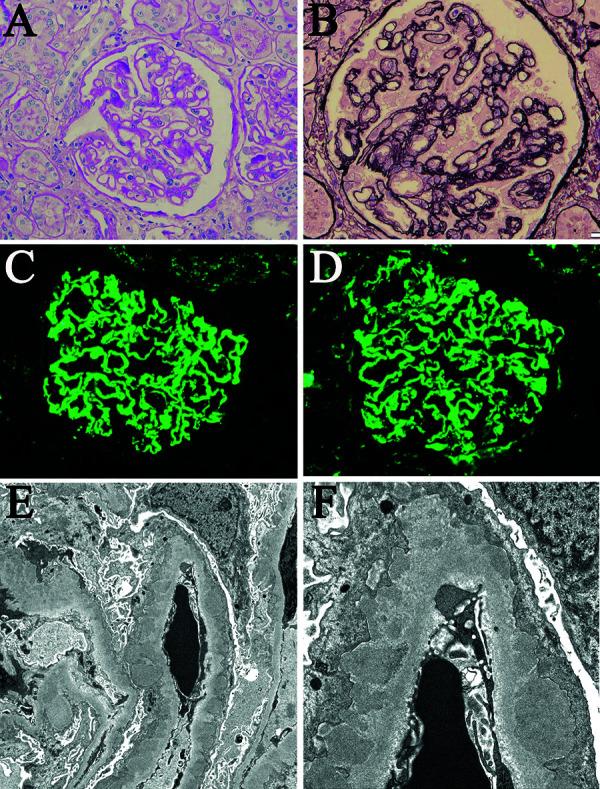
Renal biopsy findings. A: No glomerular hypertrophy or endo/extra-capillary hypercellularity was observed (periodic acid-Schiff staining, original magnification × 200). B: Clear spike lesions were detected (periodic acid methenamine staining, original magnification × 400). C, D: Immunofluorescence microscopy revealed abundant deposits of IgG (C) and C3 (D) along the capillary walls with a typical granular pattern (original magnification × 200). E, F: Electron microscopy revealed electron-dense deposits in the subepithelial region, with no deposition in subendothelial or mesangial areas (E: original magnification × 11,000; F: original magnification × 35,000).

**Figure 2.**
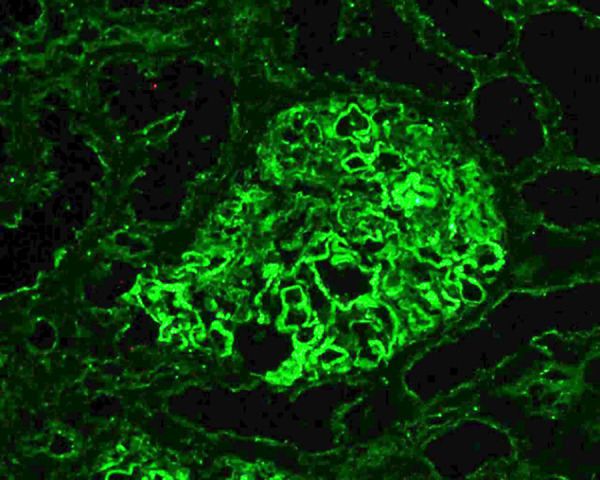
Phospholipase A2 receptor (PLA2R) staining. PLA2R staining was positive in the membranes of the capillaries, indicating PLA2R-associated membranous nephropathy (direct immunofluorescence microscopy, original magnification × 200).

Light microscopy revealed no glomerular hypertrophy or endo-/extracapillary hypercellularity (Figure 1A). PAM staining showed clear spike formation (Figure 1B). Mesangial proliferation was not observed, and there was no obvious interstitial inflammatory infiltration, including eosinophilic infiltration. Immunofluorescence demonstrated abundant IgG and C3 deposits along the capillary membranes in a typical granular pattern (Figure 1C, D). Electron microscopy identified electron-dense deposits (EDD) in the subepithelial region, but not in the subendothelial or mesangial region (Figure 1E, F). Immunofluorescence staining showed positive PLA2R staining along the capillary membranes (Figure 2). These results confirmed the diagnosis of PLA2R-associated MN. 

**Figure 3.**
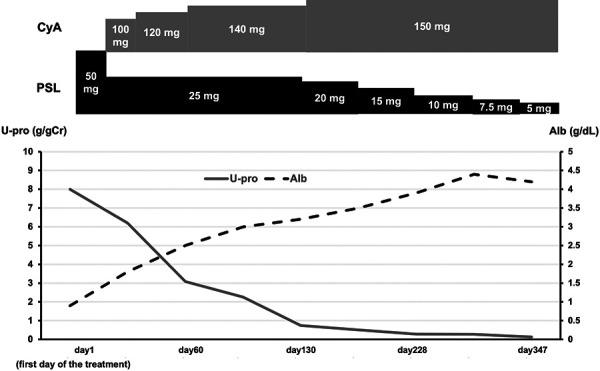
Clinical course. CyA = cyclosporine; PSL = prednisolone; U-pro = urine protein creatinine ratio; Alb = serum albumin.

The patient was initially treated with 50 mg of prednisolone (PSL) following the diagnosis of MN (Figure 3). To taper the PSL dosage, 100 mg of cyclosporine was added. After achieving remission, the PSL dosage was gradually tapered. The response to treatment was favorable, with complete remission being achieved by day 130 and maintained throughout the observation period. 

## Literature review 

We performed a literature review on 14 cases of KD with MN [3, 4, 5, 6, 7, 8, 9, 10, 11, 12, 13, 14, 15], including electron microscopy findings (Table 2). The review revealed that all cases exhibited subepithelial EDD. Of these cases, 10 did not have subendothelial EDD, while the remaining 4 lacked descriptions on subendothelial EDD. Among the 8 cases that described the mesangial region, only 2 had EDD in this area. PLA2R antigen testing was performed on 3 cases, 2 of which tested positive; however, serum PLA2R antibodies were negative in these cases. In case 11, the PLA2R antigen was negative on glomeruli, while thrombospondin type 1 domain-containing 7A (THSD7A) was positive. The target antigen was not assessed in the remaining 11 cases. 

## Discussion 

Historically, MN has been classified into primary MN (PMN), which refers to cases without associated diseases, and secondary MN, which is associated with conditions such as autoimmune diseases, infections, malignancies, or drug toxicity [16]. In 2009, Lerner et al. [18] identified PLA2R1 and its circulating autoantibodies, marking the first discovery of a podocyte-targeted antigen-antibody system in adult PMN. Approximately 70% of PMN cases are caused by circulating IgG4 autoantibodies targeting the podocyte membrane antigen PLA2R. The pathogenesis of PLA2R-associated MN is considered to proceed as follows: low levels of circulating autoantibodies against a basally expressed podocyte antigen, such as PLA2R, cross the glomerular basement membrane (GBM), bind to the antigen in situ, and form small deposits at the interface of the GBM and podocyte foot processes. Over time, these deposits develop into immune complexes, ultimately leading to podocytopathy. A key pathological feature of PMN is the formation of immune complexes along the outer surface of the GBM, adjacent to podocytes, which aligns with the proposed pathogenesis of PLA2R-associated MN described above [18]. Unlike the purely subepithelial deposits observed in PMN, secondary MN is often characterized by mesangial and subendothelial deposits in addition to subepithelial deposits [19]. These deposits form when circulating antibodies bind in situ to target antigens that have been deposited or entrapped in the mesangial and subendothelial regions, leading to the local formation of immune complexes [20]. 

Electron microscopic findings of MN in KD have only been reported in a few cases. Our literature review of MN in 14 KD cases revealed that most cases exhibited findings consistent with PMN on electron microscopy, characterized by subepithelial EDD without subendothelial EDD. These findings were consistent with those in the present case. Based on these pathological findings, the primary mechanism of MN in KD is considered to involve an antibody response to endogenous antigens on the surface of podocytes. Moreover, two-thirds of the tested cases were positive for PLA2R, suggesting that PLA2R is one of the endogenous target antigens. However, mesangial EDD was observed in 2 of 8 cases, indicating the presence of an additional pathophysiology, such as autoimmune diseases. 

If PLA2R positivity is observed and the electron microscopic findings of MN in KD are identical to those of PMN, KD may be an incidental complication of PMN. However, target antigens, such as PLA2R-, THSD7A-, or NELL1-associated MN, which are primarily observed in PMN, may also be present in secondary MN. For example, PLA2R-, THSD7A-, or NELL1-associated MN has been detected in malignancies [16]. In these cases, MN has been described by specifying the associated antigen and condition, such as THSD7A-associated MN with malignancies. Therefore, this case may be described as PLA2R-associated MN with KD. 

The present study has several limitations that need to be addressed. In our literature review, PLA2R staining was performed on only 3 of the 14 cases, with 2 testing positive. The limited sample size prevents definitive conclusions about the significance of PLA2R staining in MN with KD. Additionally, it currently remains unclear why all 3 cases with PLA2R positivity on the basement membrane, including the present case, were negative for serum anti-PLA2R antibodies. This discrepancy between antigen positivity on the GBM and undetectable serum antibodies has been reported [21]. One possible explanation is the “kidney as a sink” hypothesis, which suggests that antibodies become detectable in the serum only after the kidney’s buffering capacity is exceeded [22, 23]. Furthermore, other antigens besides PLA2R have rarely been investigated, limiting our understanding of the antigenic profile in MN with KD. In our literature review, case 11 was the only reported case in which an antigen other than PLA2R, specifically THSD7A, was examined. The author suggested a relationship between THSD7A-associated MN and allergic disorders, including KD; however, its pathogenic association with KD remains unclear. In the present case, MN developed 11 years after the initial onset of KD and 17 months following its recurrence; therefore, the possibility of a coincidental relationship cannot be excluded. Among the cases reviewed, the interval between the onset of KD and development of MN varied widely, ranging from 0 to 16 years, with a median of 24 months. Therefore, the clinical course observed in the present case does not markedly deviate from those previously reported. However, the mechanisms underlying the delayed onset of MN following KD have not yet been investigated in detail, which is a limitation of this review. 

In summary, the results of the present case and literature review suggest the involvement of autoantibodies against podocyte antigens in the pathogenesis of MN in KD. Further research is needed to identify the antigens responsible, which will provide insights into the pathophysiology of MN in KD. 

## Authors’ contributions 

MM, KK, and KY composed the initial draft of this manuscript, which was subsequently revised by all other authors, who were additionally involved in the care of this patient. 

## Funding 

The authors report no external funding source for this report. 

## Conflict of interest 

The authors have no conflict of interest to declare. 

**Table 1.** Laboratory findings on admission.

**Laboratory test**	**Results (normal range)**
Peripheral blood	
White blood cell count (/μL)	9,280 (3,300 – 8,600)
Eosinophils (%)	35 (0 – 8.5)
Red blood cell count (×10^4^/μL)	441 (386 – 492)
Hemoglobin (g/dL)	13.0 (11.6 – 14.8)
Platelet count (×10^4^/μL)	31.8 (15.8 – 34.8)
Blood chemistry	
Total protein (g/dL)	3.4 (6.6 – 8.1)
Albumin (g/dL)	0.9 (4.1 –5.1)
Lactate dehydrogenase (U/L)	245 (124 –222)
Blood urea nitrogen (mg/dL)	13.5 (8 – 20)
Creatinine (mg/dL)	0.95 (0.65 – 1.07)
Sodium (mEq/L)	141 (138 – 145)
Potassium (mEq/L)	3.7 (3.6 – 4.8)
Chloride (mEq/L)	110 (101 – 108)
Serology	
C-reactive protein (mg/dL)	0.14 (0 – 0.14)
Immunoglobulin G (mg/dL)	393 (861 – 1747)
Immunoglobulin A (mg/dL)	310 (93 – 393)
Immunoglobulin M (mg/dL)	79 (50 – 269)
Immunoglobulin E (IU/mL)	1335 (10 – 340)
Complement component 3 (mg/dL)	101 (83 – 160)
Complement component 4 (mg/dL)	28.0 (13 – 35)
50% hemolytic complement activity (U/mL)	49.4 (29 – 47)
Anti-nuclear antibody	< 40 (< 40)
Anti-PLA2R antibody	Negative
Urinalysis	
Occult blood	3+
Protein (g/gCr)	7.22
Sediment	
Red blood cells (/high-power field)	10 – 19 (dysmorphic)

PLA2R = phospholipase A2 receptor.

**Table 2.** Summary of the clinical and pathological features of cases of Kimura disease with membranous nephropathy.

**Case No.**	**Author**	**Year**	**Country**	**Sex**	**Age**	Time to onset**	**Urine protein (g/day)**	**Serum anti-PLA2R Ab**	**Glomerular PLA2R Ag**	**EDD**		
										**Sub-epithelial**	**Sub-endothelial**	**Mesangial**
1	Yamada [4]	1982	Japan	M	48	6 years	20.0	NA	NA	+	NA	NA
2	Kimura [5]	1985	Japan	M	57	1 year 6 months	0.89	NA	NA	+	NA	+
3	Akosa [6]	1991	China	M	71	0 months	3.80	NA	NA	+	–	–
4	Matsuda [7]	1992	Japan	M	68	2 years	4.00	NA	NA	+	–	–
5	Liu [8]	2008	China	M	36	1 month	14.1	NA	NA	+	–	NA
6	Liu [8]	2008	China	M	48	8 months	5.27	NA	NA	+	–	NA
7	Obata [3]	2010	Japan	M	15	13 years	2.10	NA	NA	+	–	+
8	Lee [9]	2014	Korea	F	33	1 year 6 months	16.6*	NA	NA	+	–	NA
9	Okura [10]	2014	Japan	M	22	12 years	21.0*	Negative	Positive	+	–	–
10	Gaillard [11]	2017	Switzerland	F	56	4 years	13.4	NA	NA	+	–	–
11	Hara [12]	2019	Japan	F	42	NA	0.37	NA	Negative	+	NA	NA
12	Liu [13]	2020	China	M	50	NA	NA	Negative	NA	+	NA	NA
13	Vissing-Uhre [14]	2021	Italy	M	30	16 years	13.5	Negative	NA	+	–	–
14	Yi [15]	2022	China	M	47	3 years	7.1	Negative	Positive	+	–	–
15	Present case		Japan	M	28	17 months	7.22	Negative	Positive	+	–	–

Ab = antibody; Ag = antigen; EDD = electron-dense deposits; F = female; M = male; NA = not analyzed; PLA2R = phospholipase A2 receptor; U-pro = urine protein creatinine ratio. *Proteinuria indicated by the urinary protein-to-creatinine ratio; **time to onset refers to the interval between the onset of Kimura disease and development of membranous nephropathy.
